# Calcium Signaling in Interstitial Cells: Focus on Telocytes

**DOI:** 10.3390/ijms18020397

**Published:** 2017-02-13

**Authors:** Beatrice Mihaela Radu, Adela Banciu, Daniel Dumitru Banciu, Mihai Radu, Dragos Cretoiu, Sanda Maria Cretoiu

**Affiliations:** 1 Department of Neuroscience, Biomedicine and Movement Sciences, University of Verona, Strada Le Grazie 8, Verona 37134, Italy; beatrice.mihaela.radu@gmail.com (B.M.R.); mradu@nipne.ro (M.R.); 2 Department of Anatomy, Animal Physiology and Biophysics, Faculty of Biology, University of Bucharest, Splaiul Independentei 91-95, Bucharest 050095, Romania; adela.banciu79@gmail.com (A.B.); danieldumitrubanciu@gmail.com (D.D.B.); 3 Research Beyond Limits, Dimitrie Cantemir 15, Bucharest 040234, Romania; 4 Engineering Faculty, Constantin Brancusi University, Calea Eroilor 30, Targu Jiu 210135, Romania; 5 Department of Life and Environmental Physics, Horia Hulubei National Institute of Physics and Nuclear Engineering, Reactorului 30, P.O. Box MG-6, Magurele 077125, Romania; 6 Division of Cell Biology and Histology, Carol Davila University of Medicine and Pharmacy, Bucharest 050474, Romania; dragos@cretoiu.ro; 7 Victor Babes National Institute of Pathology, Bucharest 050096, Romania

**Keywords:** interstitial cells, telocytes, calcium signaling, Ca^2+^ oscillations, pacemaker activity

## Abstract

In this review, we describe the current knowledge on calcium signaling pathways in interstitial cells with a special focus on interstitial cells of Cajal (ICCs), interstitial Cajal-like cells (ICLCs), and telocytes. In detail, we present the generation of Ca^2+^ oscillations, the inositol triphosphate (IP_3_)/Ca^2+^ signaling pathway and modulation exerted by cytokines and vasoactive agents on calcium signaling in interstitial cells. We discuss the physiology and alterations of calcium signaling in interstitial cells, and in particular in telocytes. We describe the physiological contribution of calcium signaling in interstitial cells to the pacemaking activity (e.g., intestinal, urinary, uterine or vascular pacemaking activity) and to the reproductive function. We also present the pathological contribution of calcium signaling in interstitial cells to the aortic valve calcification or intestinal inflammation. Moreover, we summarize the current knowledge of the role played by calcium signaling in telocytes in the uterine, cardiac and urinary physiology, and also in various pathologies, including immune response, uterine and cardiac pathologies.

## 1. Introduction

### 1.1. Definition and Nomenclature for Interstitial Cells

Interstitial cells are defined as cells pertaining to or situated between parts or in the interspaces of a tissue. These cells are located in the connective tissue and under the umbrella of this terminology we find reunited cells such as the interstitial cells of Cajal (ICCs), the testosterone-secreting cells of the testis (Leydig cells), the cells in the medulla and cortex of the kidney, the cells found in the connective tissue of the ovary, the aortic valve interstitial cells, etc. [1,2,3,4]. As one can perceive, all these cells differ as to origin and phenotype. Moreover, histologists consider that the usually described cells of the connective tissue might also be viewed as interstitial cells, e.g., fibroblasts, mast cells, macrophages and blood-derived immune cells (plasma cells, neutrophils, eosinophils, and lymphocytes). From the point of view of pathologists, all cells expressing vimentin can be identified as interstitial cells [5]. This review describes calcium signaling in interstitial cells with a special focus on ICCs in the gastrointestinal tract, on interstitial Cajal-like cells (ICLCs) in the extra-digestive organs and on telocytes (TCs), a novel type of interstitial cells. Several other interstitial types are reached in our discussion because the signaling through calcium oscillations is significant in their case although these cells differ in origin.

### 1.2. Interstitial Cells of Cajal (ICCs)

ICCs were described in 1892, by the Spanish neuroanatomist, histologist and pathologist, Santiago Ramon y Cajal, as primitive neurons in the intestinal wall [6]. There are many attempts to classify the ICCs as different subtypes, most of them based on their location in various organs of the digestive tract wall [7]. During the twentieth century, ICCs were described morphologically in the gastrointestinal tract as frequently forming networks around the myenteric plexus (Auerbach’s plexus) and along the whole digestive tube in the submucosa, in the connective tissue septa of the muscularis and in the subserosa. These classifications are not subject to this review.

#### 1.2.1. Ultrastructural Features of ICCs

In the mid-1970s, the ultrastructural features of ICCs were described for the first time by an Italian electron-microscopist Faussone-Pellegrini, who also resumed the first functional hypothesis postulated by Tiegs regarding the role of ICCs in triggering, propagating and coordinating the rhythmic intestinal contractile activity [8]. Since the publishing of the first guide to the identification of ICCs, it was emphasized that these cells might be classified in species- and location-dependent types [9]. ICCs are characterized by small cell bodies and several narrow, rounded, or only slightly flattened cytoplasmic extensions. The ultrastructural criteria for ICCs’ differential and positive diagnosis include: (a) the presence of a discontinuous basal lamina; (b) numerous plasmalemmal caveolae; (c) numerous mitochondria and abundant intermediate filaments, moderately developed Golgi apparatus, and rough and smooth endoplasmic reticulum cisternae and tubules in the cytoplasm; and (d) close contacts established with nerve endings and the realization of numerous gap junctions, both with each other, and with smooth muscle in the muscularis of the enteric wall [10].

#### 1.2.2. Immunophenotype of ICCs

Today, in the “omics” era, electron microscopy remains the only reliable method of identification of ICCs. Different histological techniques, starting with the ones described by Cajal himself, were shown to have their limitations in ICCs identification [11]. This fact represented a challenge for immunohistochemistry which still struggles to identify an appropriate marker for the identification of interstitial cells. In the early 2000s, antibodies to c-Kit were considered as useful to the identification of ICCs [12,13] even more as the lack of expression of proto-oncogene c-kit appeared to be closely correlated with the loss of mechanical rhythmicity of the gut [14,15]. In the late 2000s, Ano1, a Ca^2+^-activated Cl^−^ channel, was demonstrated to be a highly specific marker for studying the distribution of ICCs in the gastrointestinal tract, being able to label all classes of ICCs [16,17].

#### 1.2.3. Roles of ICCs

The acknowledged physiological roles of ICCs are: (a) to provide pacemaker activity in the gastrointestinal smooth muscles; and (b) to act as transducers of inputs from motor neurons, and stretch receptors [18]. These functions are Ca^2+^ dependent [19] since the release of Ca^2+^ from internal stores activates Ca^2+^-dependent Cl^−^ channels and participates in the regenerative potentials [20,21].

### 1.3. Interstitial Cajal-Like Cells (ICLCs)

**Figure 1:**
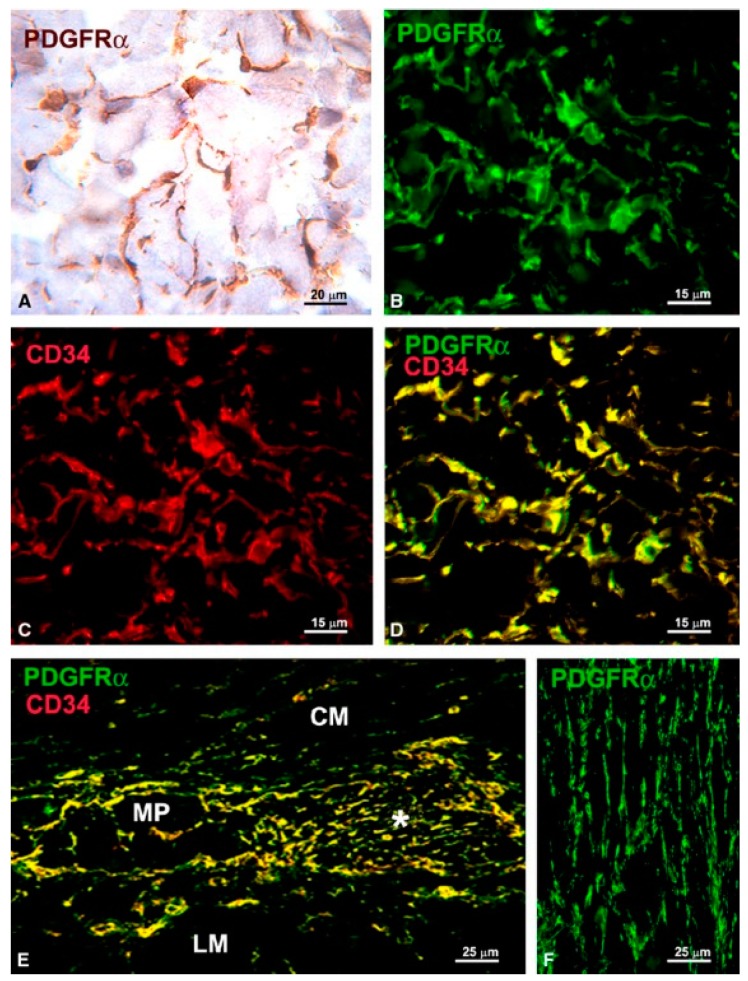
(**A**,**B**,**F**) PDGFRα-immunoreactivity; (**C**) CD34-immunoreactivity; and (**D**,**E**) PDGFRα/CD34 double labeling. (**A**) Immunohistochemistry, hematoxylin counterstain; (**B**–**F**) Immunofluorescence. (**A**–**D**) Submucosa (stomach). PDGFRα-positive cells (**A**,**B**) and CD34-positive cells (**C**) form a 3-D network. All the PDGFRα-positive cells are also CD34-positive (**D**). (**E**) Myenteric plexus region (large intestine). PDGFRα/CD34-positive cells surround a ganglion (left side, MP) and form networks in the intergangliar region (right side, asterisk). (**F**) Circular muscle layer (small intestine). PDGFRα-positive cells form networks among the smooth muscle cells. CM: circular muscle layer; LM: longitudinal muscle layer. Scale bars are indicated in each panel. Reproduced from [39], published under the Creative Commons license.

The mid-2000s were marked by the discovery of c-kit positive cells in organs capable of peristaltic movements, e.g., the upper urinary tract, bladder, and vas deferens [22,23,24]. Their role was discussed extensively in reviews at that time and it still is nowadays [25,26,27,28]. Gradually, such cells were described in several extra-digestive organs: uterus, fallopian tube, vagina, pancreas, prostate, mammary gland and blood vessels [29,30,31,32,33,34,35]. These cells, which resemble, by ultrastructural appearance, the canonical ICCs, were found at that time under different names: ICC-like cells, interstitial Cajal-like cells (ICLC), platelet-derived growth factor receptor alpha (PDGFRα) positive cells, etc. In their attempt to characterize these cells, researchers found that the interstitial cells located in the above-mentioned organs might express different immunophenotypes depending on their location in organs and on species [36,37]. In fact, while some of these cells might share morphological, immunophenotypical and functional similarities with ICCs, others share the same characteristics with TCs [38]. In a set of reports, Vannucchi and collaborators [1,38,39] solved the controversy regarding the existence of a different population of interstitial Cajal-like cells in the gut. These cells, expressing cluster of differentiation 34 (CD34) and PDGFRα in the gut, were shown to be involved in regulating motility by interaction with ICCs [39]. As shown in Figure 1, authors demonstrated that all cells expressing CD34 are also positive for PDGFRα, and therefore these cells could not be interpreted as ICCs which are diagnosed by their c-kit immunophenotype. At present, there is still controversy regarding the various classes of interstitial cells, however, the presence of PDGFRα positive cells in the gut is admitted by many groups as reviewed by Sanders et al. [40].

### 1.4. TCs

#### 1.4.1. Discovery, Definition and Ultrastructural Features

**Figure 2:**
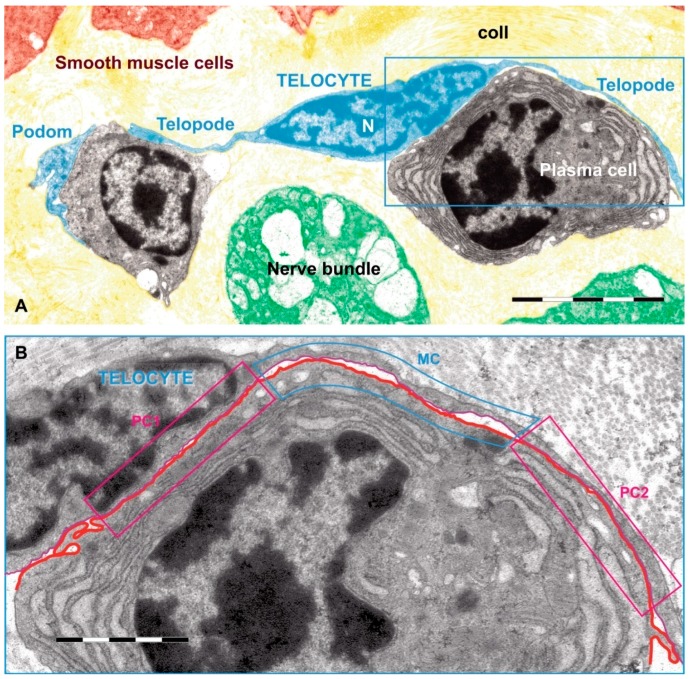
Rat jejunum mucosa: (**A**) A telocyte (blue) telopode is engaged in different types of synapses with a plasma cell, and two plain synapses (PC1 and PC2) and one multicontact synapse (MC) are seen; (**B**) region magnified from (**A**). Scale bar: (**A**): 5 µm, (**B**): 2 µm. Reproduced with permission from [44].

TCs were described as a new type of interstitial (stromal) cells in 2010 by Popescu’s team. They had started to search for ICCs in the extra-digestive organs and stopped when they realized that what they observed was, in fact, a distinct cell type [41]. TCs are shortly defined as cells with telopodes. Telopodes is the name used to describe the extremely long (tens to hundreds of micrometers) and thin (between 0.05 and 0.2 micrometers) cytoplasmic extensions emitted from the cell body [42]. In addition, telopodes are made up of a succession of thin, fibrillar segments called podomers (~75–80 nm) and the dilated, cistern-like regions called podoms (250–300 nm) [43,44]. Podoms accommodate functional units consisting of caveolae, mitochondria, and endoplasmic reticulum, possibly involved in calcium uptake/release [45]. Cytoplasmic organelles are limited, e.g., mitochondria 5%, endoplasmic reticulum 1%–2%, and caveolae 2%–3% of cell volume [41]. TCs are able to release extracellular vesicles and therefore are considered as important players in intercellular communication (for review see [46]). Telopodes are building up a 3D network by interacting with each other by homocellular junctions [45]. In addition, TCs contact, by their telopodes, numerous surrounding cells or structures. Figure 2 is relevant for such heterocellular contacts between a TC and some immune cells [44].

**Figure 3:**
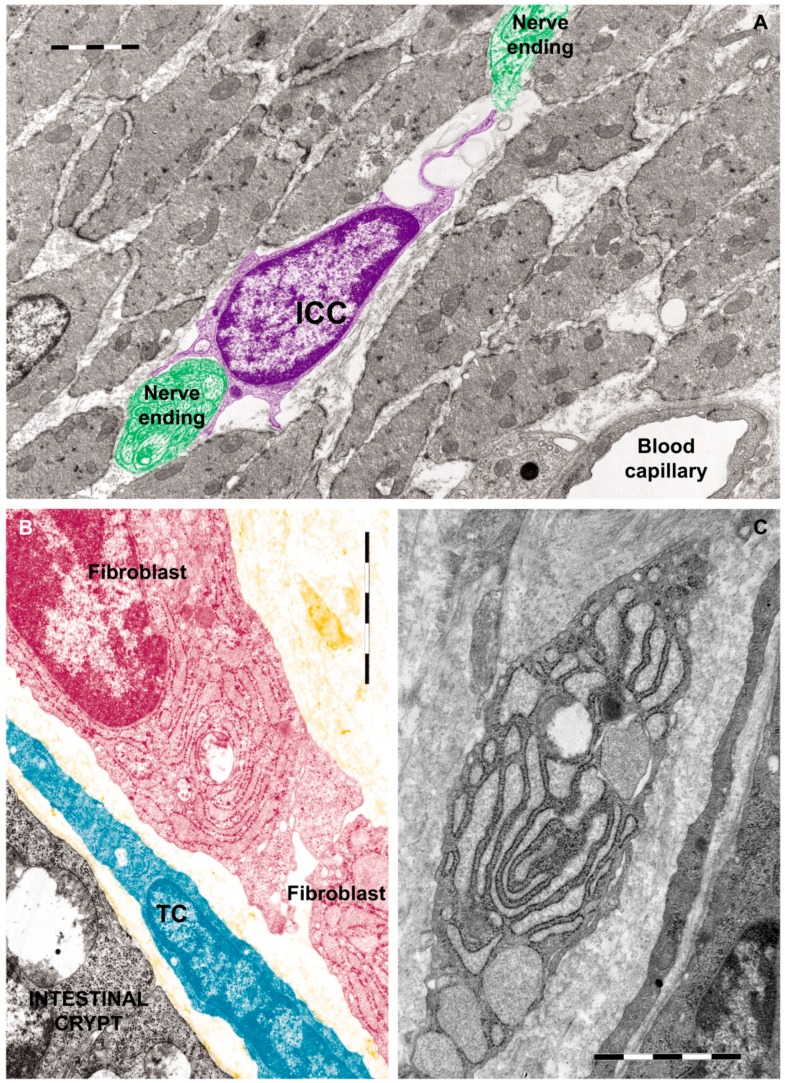
Rat jejunum: (**A**) Photomicrograph of an interstitial cell of Cajal (violet) in muscularis externa. Note the large cell body which extends a slender and relatively short connection towards the nerve endings (green); (**B**) Digitally colored TEM image showing a fibroblast (garnet) and a telocyte (blue) in the lamina propria; (**C**) Transmission electron micrograph (TEM) of a tangential section through a fibroblast cell. The internal structure can be seen, including the dilated rough endoplasmic reticulum responsible for synthesizing collagen. Reproduced with permission from [44].

There is a growing body of evidence highlighting that TCs are different from ICCs, fibroblasts (as shown in Figure 3) and mesenchymal stem cells, not only by morphology [44] but also by their genomic and proteomic characteristics [47].

#### 1.4.2. Immunophenotype of TCs

Several experiments performed by many research groups in their attempt to find a specific immunohistochemical marker illustrate that the most appropriate way to differentiate between TCs and other interstitial cells is the double-positive immunostaining with CD34/PDGFR (α or β). Additionally, TCs positive for the alpha smooth muscle antibody (αSMA) or for calreticulin antibody (calret) have also beenconsidered in this classification. Thus, in his articles Vannucchi and collaborators [1,38,39] describe in the bladder, three subtypes of TCs: the first subtype located beneath urothelium was PDGFRα/calret-positive and αSMA/CD34/c-Kit-negative; the second subtype in the deep suburothelium is PDGFRα/calret/αSMA-positive and CD34/c-Kit-negative; and a third TC subtype, PDGFRα/αSMA/c-Kit-negative and CD34/calret-positive, is in the submucosa and detrusor. Díaz-Flores et al. emitted the hypothesis that TCs have progenitor capacity and are a source of αSMA+ cells during repair [37].

#### 1.4.3. Roles of TCs

TCs were very well characterized during previous years regarding their genomic and proteomic profiles [48,49,50,51,52]. TCs are not fibroblasts or mesenchymal stem cells [53,54]. Among the most important functions of the TCs, we can mention that of integrators of many intercellular signaling processes (for details see our latest review [55]).

## 2. Calcium Signaling in Interstitial Cells

### 2.1. Main Calcium Signaling Pathways in Interstitial Cells

RT-PCR based studies proved the presence in ICCs of some specific neurotransmitter receptors assuring the functional connection of these cells to adjacent neurons. In ICCs isolated from the murine gastrointestinal tract the expression of muscarinic acetylcholine receptors (M_2_ and M_3_) and of substance P receptors were evidenced [56]. In addition, the purinergic receptor P2X (P2X2 and P2X5 subtypes) has been found by immunofluorescence in the ICCs of guinea pigs intestines [57]. Among the calcium permeant membrane channels, the transient receptor potential melastin channels, particularly TRPM7, were identified in the ICCs from the human gastro-intestinal tract [58]. Moreover, the calcium oscillations are dependent on the presence of the calcium concentration outside of the ICCs from rabbit urethra [59]. These findings clearly indicate the presence in ICCs of the most common calcium signaling pathways: the IP3 path and the store operated membrane calcium channels path. Actually, the description of the mechanism of the oscillations is based on these two basic cytosolic Ca^2+^ control modalities.

The IP3/Ca^2+^ signaling pathway was described to be involved in multiple cellular processes, including metabolism, contraction, fertilization, exocytosis, proliferation, fluid secretion, neuronal synaptic plasticity, aggregation, ion channel opening, aldosterone secretion, differentiation, proliferation, etc. [60,61]. The IP3/Ca^2+^ oscillatory mechanism controls the rhythmic contractions of vascular, lymphatic, airway and corpus cavernosum smooth muscle cells [62], and was also proposed to be active in pacemaking cells such as ICCs [61,63]. Ca^2+^ signaling mechanisms in ICCs involve both ryanodine receptors (RyR) and inositol triphosphate receptors (InsP3R). The interdependence between RyR and InsP3R in the generation of Ca^2+^ transients, and the dominant transcripts expression of Itpr1 and Ryr2 in ICCs were demonstrated [64].

A very important aspect should be highlighted: isolated ICCs present differences in Ca^2+^ signaling mechanisms with respect to the cells in intact muscles. To date, it was suggested that release of Ca^2+^ from both IP_3_ and ryanodine receptors is important in generating pacemaker activity in ICCs [65].

Another important calcium signaling pathway, based on the calcium ions influx from outside of the cells, involves the presence of the plasma membrane channels permeable for calcium. As already mentioned, TRPM7 channels have been identified by immunofluorescence in human small intestine and colon and colocalized with c-KIT, proving that ICCs have this type of calcium permeant channels in the membrane [58]. The ability of non-specific calcium influx blockers La^+3^ and Cd^2+^ to abolish the calcium oscillations in ICCs from rabbit urethra proves the involvement of the store-operated channels path in maintaining the pacemaking activity of ICCs [59]. However, other membrane channels, such as voltage-gated calcium channels [66] and calcium-activated chloride channels including ANO1 [67], are players in the calcium oscillation generation and maintenance mechanism.

Little is known about these mechanisms in telocytes. However, the T and L subtypes voltage-gated calcium channels have been found in the telocytes from human myometrium [68].

### 2.2. Ca^2+^ Oscillations in Interstitial Cells

The ICCs and ICLCs are considered as electrical pacemaker cells and this role was documented for some organs like gastrointestinal tract, urinary tract and male genital organs [63,69,70]. It is also considered that ICLCs might intermediate the signal transduction between nervous and muscle cells [71]. These types of activity are accompanied by the generation of calcium waves of specific amplitude and frequency. The calcium signaling mechanisms involved in the generation of these waves are poorly characterized but include the basic pathways involving the release of calcium from the internal stores and the subsequent calcium operated membrane channels opening [71,72,73,74,75,76]. For some particular tissues, a model describing the Ca^2+^ oscillations in ICCs was proposed. It follows the general ideas of calcium waves generation in cardiac pacemaker cells using the main Ca^2+^ controlling paths mentioned in the previous section [77], but the model has to be verified for a larger range of ICCs, particularly for TCs.

As an important issue, these Ca^2+^ signals present ubiquitous temporal characteristics depending on species and tissues and have greater amplitude but lower frequency in comparison with the signals in the neighboring smooth muscle cells [70].

For the ICCs in the gastrointestinal tract a complex calcium-dependent signaling mechanism in several steps was proposed: (i) release of Ca^2+^ through both IP_3_ and ryanodine receptors in the endoplasmic reticulum membrane; (ii) activation of ANO1 channels from the plasma membrane; (iii) current flux through ANO1 channels; (iv) spontaneous transient inward currents determine the generation of spontaneous transient depolarizations; (v) the Ca^2+^ influx is driven through T-type voltage-dependent Ca^2+^ channels; (vi) the Ca^2+^ influx promotes further Ca^2+^ release through subcellular IP_3_ and ryanodine receptors; (vii) the enhanced release of Ca^2+^ from the ER synchronizes the opening of additional ANO1 channels; (viii) a slow wave current that spreads to adjacent smooth muscle cells via gap junction proteins is generated; (ix) the slow wave current causes the smooth muscle depolarization; and, finally, (x) the contraction of the gastrointestinal wall is triggered [71,78,79].

### 2.3. Cytokines and Vasoactive agents Modulate Calcium Signaling in Interstitial Cells

Interleukin-9 (IL-9) was shown to promote proliferation of ICCs and to enhance cholecystokinin-8-induced Ca^2+^ transients [80]. Moreover, in murine gastric antral tissues, IL-9 receptor and cholecystokinin-1 receptor were co-localized with c-kit immunoreactivities [80]. It was also proved that IL-9 had a proliferative effect on ICCs inside tissue explants and that injured ICCs establish membrane-to-membrane contacts with mast cells in correlation with piecemeal degranulation at the ultrastructural level [81,82]. Additionally, mast cells were demonstrated to secrete IL-6 that modulated ICCs growth and repair [81].

Bone morphogenetic protein 2 (BMP-2) and tumor growth factor beta 1 (TGF-β1) were described to be responsible for biglycan-induced pro-osteogenic reprogramming in human aortic valve interstitial cells, to upregulate the expression of osteogenic biomarkers and consequently to stimulate calcium deposition in these cells [83]. Additionally, TNF-α accelerated the calcification of human aortic valve interstitial cells obtained from patients with calcific aortic valve stenosis via the BMP2-Dlx5 pathway [84]. Vasoactive agents, e.g., serotonin and angiotensin II, were described to elicit maximal intracellular Ca^2+^ transients in cultured human valve interstitial cells [85].

Moreover, bradykinin was identified to modulate the pacemaker activity in cultured ICCs through bradykinin B_2_ receptor activation by external Ca^2+^ influx and internal Ca^2+^ release via PKC- or cyclooxygenase-independent mechanism [86]. Histamine was also shown to act on ICCs and to modulate the pacemaker activity through H_1_ receptor-mediated pathways via external Ca^2+^ influx and Ca^2+^ release from internal stores [87].

We may conclude that, additionally to neurotransmitters, some cytokines and vasoactive molecules are involved in controlling/mediating the ICCs function.

## 3. Physiology and Alterations of Calcium Signaling in Interstitial Cells

**Figure 4:**
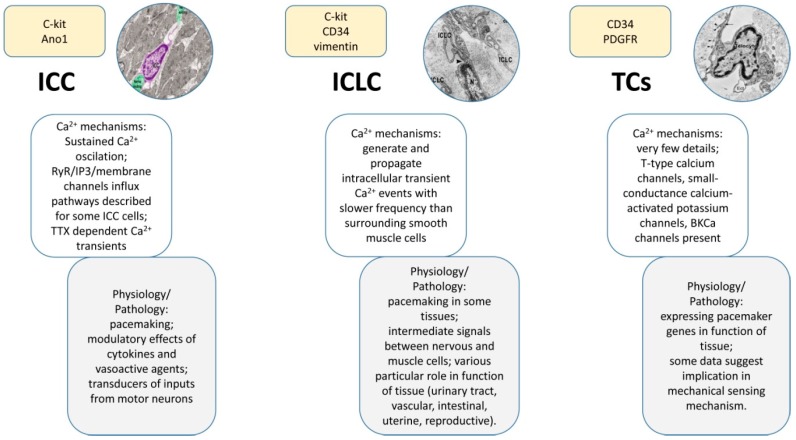
Compendious comparison of main features among the three types of cells described here: ICCs, ICLCs, and TCs. Knowledge about calcium signaling and related physiological processes are highlighted. As expected, the ICCs are the best described and the TCs the poorest. One can observe certain similarities between the ICLCs and the other two types of cells, explained by the fact that both cell populations co-exist under this acronym. Insets reproduced with permission from [44,157].

Calcium signaling pathways have been described in various subtypes of interstitial cells (Table 1), including ICCs, ICLCs, TCs, valve interstitial cells or Leydig cells, and the main anatomical systems have been targeted (e.g., urinary, cardiovascular, gastrointestinal, reproductive system, etc.). This exhaustive analysis revealed the contribution of the interstitial cells to the physiology and pathology of these systems. Besides the importance of calcium signaling in interstitial cells to the pacemaking activity (Figure 4), it should be emphasized that the intracellular Ca^2+^ fluctuations are also contributing to a wide range of physiological and pathological roles played by interstitial cells, including reproductive function, tissue remodeling, immune signaling, mechanical sensing, etc.

### 3.1. Physiological Role of Calcium Signaling in Interstitial Cells

#### 3.1.1. Calcium Signaling in Gastrointestinal Interstitial Cells

The intestinal ICCs have been described to be electrically coupled to smooth muscle cells [18,88] and to contribute to the pacemaking activity by affecting the resting membrane potential of the smooth muscle cells [89]. Recent studies have described the Ca^2+^-associated mechanisms in the intestinal ICCs that are contributing to the intestinal pacemaker activity. To date, the gastrointestinal distension (e.g., hypotonic stress) induces sustained inward holding current via actin microfilaments and the process is mediated by alteration of intracellular basal Ca^2+^ concentration and Ca^2+^ oscillations in murine cultured intestinal ICCs [90]. Ca^2+^ oscillations in gastrointestinal ICCs were described to depend on Ca^2+^ influx mediated by transient receptor potential-like channel 4 (TRP4) in caveolae [91].

Although ICCs have been generally accepted as being able to tune the luminal chemical environment, that prepares the membrane potential of smooth muscle cells (SMCs) for depolarizing or hyperpolarizing response, recent studies provide evidence for the non-contribution of ICCs to the enteric inhibitory neuromuscular neurotransmission [92].

#### 3.1.2. Calcium Signaling in the Interstitial Cells of the Urinary Tract

ICCs act as pacemakers in the urinary tract [93]. ICCs were identified both in the upper and lower urinary tract, and voltage clamp recordings indicated that these cells present abundant calcium-activated chloride currents and spontaneous transient inward currents blocked by chloride channel antagonists [93]. Depending on the segment of the urinary tract, ICCs may act or not as pacemaker cells. In the urethra, ICCs have been considered as “loose pacemakers” that provide multiple and randomly modulatory inputs to the smooth muscle cells, while in the bladder or the renal pelvis, these cells are only modulators of the smooth muscle activity [94]. Other studies indicated that ICCs from the urethra are specialized pacemakers involved in the generation of urethral tone and in the maintenance of urinary continence, and there is an important contribution of intracellular Ca^2+^ stores and Ca^2+^ influx to these pacemaking mechanisms [74].

Stimuli, e.g., caffeine, muscarinic or purinergic agonists, elicit intracellular calcium concentration increases in isolated c-kit positive cells from the suburothelial layer [74,95]. Moreover, experiments with neurogenic electrical field stimulation of guinea-pig bladder tissue samples indicated that all subtypes of ICCs and smooth muscle cells displayed in situ spontaneous Ca^2+^ transients that were tetrodotoxin-sensitive [96].

In mouse preparations of the ureteropelvic junction, membrane depolarization of stellate ICLCs evoked a slowly developing outward current that did not arise from the opening of transient outward current or large conductance Ca^2+^-activated K^+^ currents [97]. Whole-cell current-clamp recordings on ICLCs showed random fluctuations of membrane potential and occasionally large, long-lasting depolarizations, while voltage-clamp recordings showed high-frequency spontaneous transient inward currents, and the authors concluded that ICLCs could contribute to the ureteropelvic junction pacemaking in the absence of a pacemaker drive [97]. ICLCs from the lower urinary tract have been described to generate and propagate intracellular transient Ca^2+^ events [71].

However, the details of this Ca^2+^ spikes occurrence are still not well described, and a possible role of neuronal triggering can be considered since the ICCs react by means of calcium responses to the presence of external neurotransmitters.

#### 3.1.3. Calcium Signaling in Interstitial Cells of the Female Reproductive System

Oxytocin-induced [Ca^2+^]i oscillations have been demonstrated in primary cultures of human uterine myocytes [98,99], but data recorded in intact cells residing within the myometrium are limited. However, a recent study on organotypic slices from human myometrium indicated that oxytocin-induced [Ca^2+^]i oscillations occurred only in a proportion of cells and were not relevant for the acute regulation of myometrial contractility, but the authors suggested the involvement of [Ca^2+^]i oscillations in long-term regulatory processes, e.g., gene expression triggering [100]. These cells were identified to be ICCs or ICLCs and described to be morphologically and phenotypically distinct by SMCs.

To date, the contribution of ICCs and/or ICLCs to the pacemaking activity was analyzed in different segments of the reproductive system. In murine oviducts, ICCs were described as pacemakers being responsible for generating slow waves underlying myosalpinx contractions that are critical for egg transport [101]. Additionally, calcium imaging of live tissue slices from myometrium show that ICLCs located on the edge of the smooth muscle bundles initiate the contractile wave [43]. Moreover, ICLCs from myometrium exhibit in vitro spontaneous electrical activity characterized by membrane potentials of 62.4 ± 7.22 mV, and short duration: 1.197 ± 0.04 ms [29]. More systematic experiments are necessary to confirm a detailed picture of calcium activity in the reproductive system ICCs.

#### 3.1.4. Calcium Signaling in Interstitial Cells of the Male Reproductive System

Leydig cells are a subtype of interstitial cells that have been described in the seminiferous tubules of the testicles. BKCa channels were characterized to be activated by the increase of the intracellular Ca^2+^ and to determine the cell membrane hyperpolarization. In Leydig cells, the hyperpolarization induced by the BKCa channels was speculated to activate a series of events that limits testosterone production [102]. Oppositely, hormones might regulate the intracellular Ca^2+^ concentration in Leydig cells. To date, the luteinizing hormone was shown to modulate the T-type calcium channels and the intracellular Ca^2+^ transients through PKC and PKA signaling pathways, and thus, these kinases, besides the direct action to promote testosterone synthesis, also act on the overall calcium dynamics in Leydig cells [103]. Mibefradil was shown to inhibit T-type calcium channels in Leydig cells and steroidogenesis is linked to the Ca^2+^ entry through the T-type Ca^2+^ channel [104].

Exposure of Leydig cells to polychlorinated naphthalenes increased the intracellular Ca^2+^ concentration, the sex steroids production and the mRNA expression of estrogen-related receptors α, β and γ [105]. Several studies pointed out that the Ca^2+^ molecular pathways are essential for steroidogenesis in Leydig cells and that the transcriptional cascade involving the nuclear receptor NR4A1 regulates steroidogenesis [106,107].

#### 3.1.5. Calcium Signaling in Interstitial Cells from the Vascular System

Vascular interstitial cells from the portal vein were shown to play an important role in the rhythmic vascular activity that contributes to the vascular tone [108]. In particular, mitochondrial Ca^2+^ is essential for the generation of the rhythmic Ca^2+^ waves in vascular interstitial cells [108].

Vascular interstitial cells were discussed to be similar to the previously described ICCs and ICLCs [35,109,110] and were characterized in a large variety of blood vessels preparations (e.g., rabbit portal vein and mesenteric and cerebral arteries; rat portal vein, aorta and pulmonary, mesenteric, kidney, coronary and cerebral arteries; mouse aorta, mesenteric and cerebral arteries; guinea pig cerebral, portal vein, mesenteric and kidney arteries; and human mesenteric and gastro-omental arteries) [35,111,112,113,114,115,116]. It should be emphasized that in primary vascular cell cultures, vascular interstitial cells display slow rhythmical changes of the intracellular Ca^2+^ concentration, while contractile vascular smooth muscle cells present faster Ca^2+^ sparks [109], and that both subtypes of Ca^2+^ signals are generated close to the apposition between the perinuclear Ca^2+^ store and endoplasmic reticulum network [110,117].

### 3.2. Pathological Role of Calcium Signaling in Interstitial Cells

#### 3.2.1. Modulation of Calcium Signaling Pathways in Interstitial Cells as a Therapeutic Strategy against Aortic Valve Calcification and Aortic Stenosis

Valve interstitial cells have been extensively studied. The comparison between the transcriptional profiles and cellular functions of the human aortic valve interstitial cells and mitral valve interstitial cells indicated expression differences for seventy-eight genes, among those *NKX2–5*, *TBX15*, *OGN*, *OMD*, and *CDKN1C* having a higher expression and *TBX5*, *MMP1*, and *PCDH10* a lower expression in aortic valve interstitial cells [118]. Interestingly, mitral interstitial cells proliferated more quickly and showed more calcium deposition and alkaline phosphatase activity than aortic interstitial cells [118].

Calcific aortic valve disease is a slowly progressive disorder that ranges from aortic sclerosis to severe calcification, with multiple microscopic characteristics, including endothelial damage and lipid deposition. Valvular interstitial cells are thought to be involved in tissue remodeling and repair during the cyclic movement and mechanical stress of aortic valves [119]. Valvular interstitial cells are located on the internal side of the heart valves, being different between the three cusps, and only a subpopulation of these cells are predisposed to calcification [120].

In aortic valve interstitial cells, rapamycin, a commonly used immunosuppressant, was described to inhibit Toll-like receptor 4 (TLR4)-induced osteogenic responses by activation of signal transducer and activator of transcription 3 (Stat3) through Akt, and to alleviate the inflammation-induced initiation and progression of calcific aortic valve disease [125].

Denosumab, a human monoclonal antibody that binds the receptor activator of nuclear factor κ-β ligand, was shown to reduce calcium deposition in the aorta [126], but the mechanism by which it affects ectopic calcification was poorly understood in the last decade. A recent study highlighted that denosumab may act as an in vitro inhibitor of valvular interstitial cells calcification [127].

#### 3.2.2. Interstitial Cells Dysfunction during Intestinal Inflammation

ICCs play an important role in the gastrointestinal inflammation. Inflammation-induced alterations in the network of ICCs from the small intestine associated with Auerbach’s plexus lead to gastrointestinal motility disturbances [128]. Recently, it was demonstrated that during the intestinal inflammation, nitric oxide-induced oxidative stress impaired the pacemaking function of murine ICCs [129]. Indeed, treatment of ICCs with interferon-γ and lipopolysaccharides for 24 h reduced the frequency and the amplitude of calcium oscillations in these cells [129]. However, the possibility of a direct action of cytokines on ICCs during the inflammatory process could be another explanation.

## 4. Calcium Signaling in TCs

TCs are ubiquitous cells localized in the various mammalian anatomical structures, e.g., cardiovascular, respiratory, digestive, reproductive, urinary, musculoskeletal, integumentary, visual, nervous, and hematopoietic systems [130]. However, the study of calcium signaling in TCs is a recently opened research direction. The pioneering studies done so far have been rather focused on the description of calcium channels at the plasma membrane level by immunohistochemistry and electrophysiology techniques. This topic has many uncovered aspects to be further investigated. Actually, there are no papers reporting direct evidence of calcium transients in TCs. In the following paragraphs, we review the very little information reported until now on this topic.

### 4.1. Physiological Role of Calcium Signaling in TCs

#### 4.1.1. Contribution of Calcium Signaling in TCs to the Uterine Physiology

A novel class of PDGFR-α(+) interstitial cells was described in mouse and monkey female reproductive tracts. It is distinct from smooth muscle cells and ICCs, and was characterized to have a variable gene expression between parts of the reproductive tract (e.g., ovary, oviduct, and uterus) or between the tissue regions of the same organ (e.g., uterine myometrium vs. endometrium) [131]. These cells are unlikely to provide pacemaker activity, as key pacemaker genes found in ICCs (e.g., *Kit*, and *Ano1*) [121,132] were not detected to be expressed, while the *Gja1* gene encoding for connexin 43 was identified in high levels suggesting their possible involvement in forming gap junctions in between and with the neighboring smooth muscle cells [131]. CD34 and PDGFRα are considered as reliable markers to identify and separate TCs [47,133,134] and we might suppose that the newly identified interstitial cells are corresponding to TCs.

Recent studies have described by immunohistochemistry and in vitro electrophysiology the presence of T-type calcium channels in cultured human myometrial TCs [68,123]. Cretoiu et al. reported difficulties in recording T-type calcium channels in human myometrial TCs by applying the standard patch-clamp protocol consisting in step depolarization pulses from −90 to +40 mV of 100 ms duration, 10-mV increment from a holding potential of −110 mV [68]. However, the authors succeeded to activate T-type calcium currents in human myometrial TCs by applying a brief depolarizing ramp protocol from −90 to +60 mV with a duration of 100 ms and steepness of 1.5 V/s [68]. Mibefradil, a selective inhibitor of T-type calcium channels, was demonstrated to block these currents in uterine TCs [68]. Additionally, acute (30 min) and chronic (24 h) exposure of TCs from pregnant myometrium to mibefradil determined a significant reduction in the low-level laser stimulation telopodal lateral extension growth rate [123].

TCs are considered as mechanical sensors in human uterus, probably being involved in the detection and translation of the stretch stimuli to the nuclear factors and in the activation of genes encoding protein synthesis [135,136]. We might suppose that calcium signaling in uterine TCs plays an important role in the mechanical sensing mechanism, as mibefradil was already shown to distinctly modulate TCs sensitivities from nonpregnant and pregnant myometrium to low-level laser stimulation [123]. As TCs from pregnant myometrium were more susceptible to deviate the growth direction of telopodal lateral extension than those from nonpregnant myometrium when exposed to low-laser laser stimulation [123], then these cells were proposed to play an important role in the uterine contraction mechanism in a direct relationship with the pregnancy status [136].

The physiological role of calcium signaling in TCs should be considered in an extended perspective as multiple ion channels are calcium-activated and/or calcium-dependent. In this context, previous studies have also reported the functional expression of hyperpolarization-activated chloride inward current with calcium dependence [122] and of small-conductance calcium-activated potassium (SK3) channels [137] in cultivated TCs from human myometrium. Interestingly, uterine TCs, but not uterine smooth muscle cells, were demonstrated to express SK3 channels, and that this expression is higher in nonpregnant compared to pregnant myometrium [137]. Moreover, SK3 activators were proposed to reduce contractility in human myometrium by modulating TCs function [137]. NS4591 (4,5-dichloro-1,3-diethyl-1,3-dihydro-benzoimidazol-2-one) is also a modulator of the calcium-activated potassium channels and was demonstrated to exert a relaxant effect on the human myometrial spontaneous contractility in vitro [138].

#### 4.1.2. Contribution of Calcium Signaling in TCs to the Cardiac Physiology

Oppositely to the uterine TCs that do not express the pacemaker-related *Kit* and *Ano1* genes [131], cardiac TCs express CD34, CD29, vimentin, sca-1, c-kit, and Nanog, and are more likely to be involved in the heart pacemaking activity [133,139,140]. Additionally, cardiac TCs were demonstrated to functionally express large conductance Ca^2+^-activated K^+^ currents (BKCa) and inwardly rectifying K^+^ currents, but not transient outward K^+^ currents or ATP-sensitive potassium current [124]. The presence of BKCa channels in cardiac TCs strongly supports the involvement of these cells in the cardiac pacemaking activity, as previous studies have demonstrated that BKCa channels regulate sinoatrial node firing rate and cardiac pacing in vivo [141].

#### 4.1.3. Contribution of Calcium Signaling in TCs to the Urinary Physiology

Several subtypes of TCs have been described in the human urinary bladder: (i) TCs from the sub-urothelium were PDGFRα/calret-positive and CD34/c-Kit-negative, being subdivided in αSMA-negative if located immediately beneath the urothelium, and αSMA-positive when located deeper and having a larger body; and (ii) TCs from the submucosa and detrusor were PDGFRα/αSMA/c-Kit-negative and CD34/calret-positive [38]. The authors mentioned that no cell possessing the ICCs features was detected, while TCs were organized in a thick multilayered area parallel to the urothelial surface [38]. Despite the characterization of TCs in the urinary system or the already described role of Ca^2+^ signaling contribution to the urethral tone and urinary continence maintenance in ICCs and ICLCs [71,74,93,96,97], no studies have described so far the calcium signaling mechanisms in TCs from different segments of the urinary system.

### 4.2. Pathological Role of Calcium Signaling in TCs

#### 4.2.1. Contribution of Calcium Signaling in TCs to the Immune Response

Uterine TCs were described as functional players in the activation of peritoneal macrophages. Macrophages exposed to TC conditioned media contained abundant pseudopodia and cytoplasmic secretory granules without cell viability changes, and had an increased release of TNF-α, IL1-R1, and IL-10, but not TGF-β1, IL-1β, IL-23α, and IL-18 [142]. Based on the in vitro data, it was suggested that TCs are involved in the immune response, being important actors in the immunoregulatory and immunosurveillance processes [142].

Additionally to the already known involvement of the Langerhans cells, dermal dendritic cells, inflammatory dendritic epidermal cells and plasmacytoid dendritic cells in the chronic skin inflammatory process that characterizes psoriasis, recently, dermal TCs were described to be one of the important triggers for the characteristic vascular pathology in psoriasis [143]. Previous studies in psoriasis indicated alterations of the calcium metabolism in several cellular systems, and, consequently, we might suppose that TCs, as part of the immune system activation, undergo significant changes in calcium signaling changes. To date, cultured psoriatic keratinocytes present a down regulation of the capacitive calcium influx and a defective calcium-mediated cell signaling [144]. In a model of psoriasis-inflamed skin, it was proved that store-operated calcium entry proteins, e.g., stromal interaction molecule (STIM1), contribute to neutrophil chemotaxis and infiltration [145]. Moreover, there is a strong positive association between psoriasis and an increased coronary calcium score, mainly in patients with severe psoriasis [146] and hypocalcemia is considered as a risk factor in this pathology [147].

#### 4.2.2. Correlations between Elements that Modulate TCs Migration and Various Pathologies

A recently proposed theoretical model described TCs migration in chemical, metabolic and heat gradients [55]. Increased metabolism can be found in pathological cases such as tumors [148] or infections [149], but also in tissues with increased metabolic activity, e.g., striated muscle [150], cardiac muscle [151] or myometrium in different physiological [152] or pathological states [153].

Involvement of TCs signaling through calcium and increased metabolism limits the use of TCs calcium channel modulators in the pathologies affecting the heart and uterine muscles, due to a relatively uniform signaling pathway through calcium in these organs.

#### 4.2.3. TCs and Possible Roles of Calcium Metabolism in Uterine Pathologies

Modulation of TCs’ growth by mechanical factors via calcium channels has a degree of mechanical sensitivity [123] and correlates with the TCs’ ability to communicate through gap junctions [40,46], the calcium involvement in myometrium proliferation [154,155] and contraction [100]. Therefore, we propose the hypothesis that TCs contribute to smooth muscle growth in areas with high mechanical forces. This signaling mechanism may be involved in the uniform growth of uterus thickness during pregnancy by signaling the increase in thickness of the uterus in all areas with a thinner uterine wall compared with adjacent areas. This uniform growth is also modulated by normal uterine contractions. Promising therapeutic outputs include the prevention of uterine ruptures secondary to uterine hypotrophy, by employing calcium channels modulators acting on TCs. Implantation of the placenta leads to its development into the uterine wall thickness. This process decreases the thickness of the myometrium in the area with the maximum development of the placenta. This increases the mechanical forces generated by the mass of the fetus and myometrium physiological contractions in these localized areas. TCs may be involved in these areas with a compensatory increase in the thickness of the uterus, through the mechanism described above. It opens up new therapeutic opportunities for the treatment of pathological implantation of the placenta.

#### 4.2.4. TCs and Possible Roles of Calcium Metabolism in Cardiac Pathologies

Concentric muscle growth of the cavitary organs modulated by mechanical forces and calcium through the mechanism described above could justify various forms of cardiac hypertrophy (concentric and eccentric) by correlations between mechanical forces, the degree of local fibrosis that could decrease the stimulation of TCs through stretching and the degree of perfusion that is needed to hypertrophy. This theoretical model explains the link between high cardiac fibrosis after myocardial infarction and functional recovery [156].

## 5. Concluding Remarks and Perspectives

To summarize, comparing the role of calcium signaling mechanisms in different subtypes of interstitial cells to the pacemaking activity (Figure 4), we conclude that while ICCs and ICLCs are contributing to the initiation and propagation of the Ca^2+^ oscillations, TCs are not pacemakers but modulate the activity of the surrounding cells. However, this conclusion has to be confirmed by future reports on calcium signaling mechanisms, especially in the case of TCs.

Moreover, even if the role of ICCs to intermediate the information transmission from neurons to surrounding muscles cells is largely accepted, the possibility that neurotransmitters (able to trigger the cascades controlling Ca^2+^ in ICCs) are involved in the peacemaking activity is very little explored. We consider that this hypothesis could be tested in future works.

Although the existence of TCs signaling mechanisms with neighboring cells using signaling through calcium is incompletely understood, these cells open great therapeutic opportunities especially for predominantly muscular organs, which have a high degree of uniformity in the response to calcium. Muscle growth control mechanisms depending on TCs’ ability to respond to mechanical stimuli mediated by calcium channels were highlighted. Targeting these channels of TCs can lead to the development of innovative therapies for diseases with significant social and economic impacts, such as uterine pathologies and abnormal implantation of the placenta, or cardiac pathologies like hypertrophy and response to myocardial infarction. For this goal, it is necessary to develop viable ways for TCs’ separation from biological samples to allow calcium channel targeted overexpression, or expression of calcium channels modified to respond to specific drugs. Such an approach would reduce potential side effects of low specificity calcium channel modulators.

## Figures and Tables

**Table 1:** Calcium signaling in interstitial cells. Calcium signaling mechanisms in interstitial cells are described and their contribution to the pacemaking activity is indicated. The main interstitial cells involved in the pacemaking activity of different anatomical systems are ICCs and ICLCs, while TCs are not able to act as pacemakers. Other subtypes of interstitial cells (e.g., valve interstitial cells or Leydig cells) have not been described to play a role in pacemaking activity.

Anatomical Localization of the Interstitial Cells	Subtype of Interstitial Cells	Calcium Signaling Mechanisms	Contribution to the Pacemaking Activity	Reference
Gastrointestinal system	ICCs	ICCs are coupled with SMCs and affect their resting membrane potential; gastrointestinal distension induces sustained inward holding current via actin microfilaments and the process is mediated by changes in the intracellular basal Ca^2+^ concentration and Ca^2+^ oscillations in ICCs	Yes	[63,90]
Urinary system	ICCs	ICCs present abundant calcium-activated chloride currents; ICCs contribute to the urethral tone and the maintenance of urinary continence, and there is an important contribution of intracellular Ca^2+^ stores and Ca^2+^ influx to these mechanisms; ICC and SMCs display in situ spontaneous tetrodotoxin-sensitive Ca^2+^ transients	Yes/No (Depending on the segment of the urinary tract)	[74,93,96]
	ICLCs	membrane depolarization of ICLCs evokes slowly developing outward current but not the opening of transient outward current or large conductance Ca^2+^-activated K^+^ currents; ICLCs from the lower urinary tract have been described to generate and propagate intracellular transient Ca^2+^ events	Yes	[71,97]
Reproductive system	ICCs	oviduct ICCs generate slow waves underlying myosalpinx contractions that are critical for egg transport	Yes	[101]
	ICLCs	myometrial contractile signaling, associated with Ca^2+^ intracellular transients, starts on the borders of smooth muscle bundles where ICLC are located; myometrial ICLCs present in vitro spontaneous electrical activity	Yes	[29,43]
	TCs	TCs do not express key pacemaker genes (e.g., *Kit*, *Ano1*); T-type calcium channels were described to contribute to the mechanical sensing of TCs; hyperpolarization-activated chloride inward currents with calcium dependence and small-conductance calcium-activated potassium currents were described in TCs	No	[68,121,122,123]
	Leydig cells	the hyperpolarization induced by the BKCa channels was speculated to activate a series of events that limits testosterone production; steroidogenesis is linked to the Ca^2+^ entry through the T-type Ca^2+^ channel	N/A	[102,103,104,106,107]
Cardiovascular system	Vascular interstitial cells	vascular interstitial cells (similar to ICCs/ICLCs) display slow rhythmical changes of the intracellular Ca^2+^ concentration that imply both the contribution of the perinuclear Ca^2+^ store and endoplasmic reticulum network	Yes	[110,113,117]
	TCs	vascular TCs express large conductance BKCa and inwardly rectifying K^+^ currents	Probably yes	[124]
	Valve interstitial cells	valve interstitial cells are involved in tissue remodeling and repair during the cyclic movement and mechanical stress of aortic valves, but in pathological conditions are predisposed to calcification	N/A	[120]
